# Health-related quality of life in patients with obstructive sleep apnea – a systematic review update assessing the quality of measurement properties of patient-reported outcome measures

**DOI:** 10.1007/s11325-026-03676-1

**Published:** 2026-04-21

**Authors:** Alina Wildenauer, Christoph Schöbel, Torsten Eggert, Marcel Braun

**Affiliations:** Centre for Sleep and Telemedicine, University Medicine Essen, Ruhrlandklinik – West German Lung Centre, Essen, Germany

**Keywords:** Health-related quality of life, Obstructive sleep apnea, COSMIN methodology, Systematic review, Patient-reported outcome measures, Measurement properties

## Abstract

**Purpose:**

The purpose of this systematic review update was to identify existing health-related quality of life (HRQOL) patient-reported outcome measures (PROMs) for obstructive sleep apnea (OSA). Building upon a 2016 systematic review, which recommended four PROMs with the highest evidence, this investigation was warranted to synthesize new evidence and evaluate new PROMs that have emerged since then via the Consensus based Standards for the Selection of Health Measurement Instruments (COSMIN).

**Methods:**

We searched Medline via PubMed, Embase and the Cochrane Library for HRQOL PROM development and validation studies published in English or German. Studies focusing on cross-cultural validation, those in which PROMs were assessed as secondary outcomes, and studies limited to specific demographic subgroups or single HRQOL domains were excluded. For risk of bias and quality of measurement properties (MP) assessment, we used the COSMIN methodology to facilitate comparability with the previous review, as well as the GRADE approach for evidence level evaluation.

**Results:**

We identified two new PROMs, the Patient-Reported Apnea Questionnaire (PRAQ) and the Symptoms, Tiredness, Alertness, Mood and Psychosocial instrument (STAMP), which were validated in two studies and a single study, respectively. Overall, the PRAQ displays an moderate risk of bias with excellent quality of MP and high evidence for content validity in particular, whereas the STAMP shows a higher risk of bias and low quality of MP.

**Conclusion:**

The PRAQ has demonstrated the strongest evidence among PROMs since 2016. In total, including the previous review, five PROMs are currently recommended for assessing HRQOL in individuals with OSA.

**Review registration:**

The review was registered with PROSPERO under the registration number CRD42024602020.

**Supplementary Information:**

The online version contains supplementary material available at 10.1007/s11325-026-03676-1.

## Introduction

The concept of quality of life (QOL) has gained increasing importance in sleep medicine and research. Its ability to assess obstructive sleep apnea (OSA), the most prevalent sleep-related breathing disorder affecting between 30 and 50% of the world’s population [1], is pivotal. OSA is characterized by repeated collapse of the upper airway and its resulting obstructive events. Obstructions are commonly associated with oxygen desaturation and can lead to arousal reactions and sleep fragmentation [2]. Typical daytime symptoms include excessive daytime sleepiness, fatigue, and emotional and cognitive impairment, while snoring, difficulty falling and staying asleep and shortness of breath are the most common nighttime symptoms [2]. Various comorbidities are associated with OSA, such as hypertension, coronary heart disease, myocardial infarction, arrhythmias and an increased risk of stroke [3, 4]. The apnea–hypopnea index (AHI) is a metric used to gauge the severity of OSA. By counting the number of apneas and hypopneas that are present during an hour of sleep, OSA can be classified into three categories: mild (AHI ≥ 5- < 15/h), moderate (AHI ≥ 15- < 30/h) and severe (AHI ≥ 30/h) [5].

While the AHI is a core determinant for the diagnostic and follow-up treatment of OSA, it has a poor correlation with patient-reported QOL [6, 7]. This means that patients with severe OSA may report little QOL impairment, and those whose AHI improves during treatment may not experience better QOL. A comprehensive systematic review conducted in 2016 by Abma et al. [8] aimed at evaluating the quality of patient-reported outcome measures (PROM) for OSA, of which a subsection focused on health-related quality of life (HRQOL) instruments. Most items, such as the Functional Outcome of Sleep Questionnaire (FOSQ) [9] and its short version, the FOSQ-10 [10], which are frequently used in the German-speaking area, are rated as moderate to low quality. Some were of higher quality, mainly due to strong content validity, which is considered the most important measurement property (MP) according to the Consensus-based Standards for the Selection of Health Measurement Instruments (COSMIN) [11], where MP refers to a set of quantifiable characteristics that describe an instrument's quality, accuracy, reliability, and validity in measuring a construct. PROMs with good content validity include the Calgary Apnea Quality of Life Index (SAQLI) [12], the Quebec Sleep Questionnaire (QSQ) [13], the Maugeri Obstructive Sleep Apnea (MOSAS) [14] and the Obstructive Sleep Apnea Patient-Oriented Severity Index (OSAPOSI) [15], which are recommended for the evaluation of HRQOL in patients with OSA by the authors.

Despite the abundance of QOL tools, a concrete definition of the term remains ambiguous. In the first half of the twentieth century, medicine focused primarily on mortality and the prolongation of life, not necessarily on patients’ wellbeing. The term QOL [16] was first mentioned in the 1960 s in the context of emerging technologies [17]. Soon after, the term HRQOL replaced QOL in medical research, yet a core problem remains the lack of a consensual and comprehensive framing, even though the World Health Organization proposed a commonly cited definition of QOL [18]. HRQOL often includes disease-specific dimensions such as physical symptoms or therapy emergent issues but also dimensions directly affected by health, e.g., social withdrawal due to disease-induced fatigue or pain. Some conceptional models [19, 20] and definitions have been introduced that differentiate between QOL and HRQOL. While HRQOL has evolved inductively over time with varying definitions— from health’s impact on living a good life to utility values in health assessment— its conceptual boundaries with general QOL and health status, remain blurred despite extensive research [21–24]. The measurement of these latent constructs usually relies on PROMs, which the US Food and Drug Administration defines as direct patient reports of health conditions without clinical interpretation [25]. Given this unique ability to capture disease-specific emotional, social, and functional impacts alongside symptom burden, PROMs have become indispensable tools for assessing HRQOL in patient-centric care.

As demonstrated, QOL and HRQOL research continues to face considerable conceptional and methodological issues. Aiming at a working definition based on previous research, we view *QOL* as a multifactorial and subjective assessment of life’s facets, incorporating, but not necessarily being limited to, social, emotional, physical, functional and spiritual dimensions. *Health* is a person's objective and verifiable state of health, e.g., via the use of biomarkers. *HRQOL* captures how a condition affects particular QOL domains, reflecting the dynamic interplay between objective health status and subjective well-being. Each domain can influence and be influenced by others. In OSA, metrics such as the AHI or nocturnal oxygen saturation represent disease-specific health status, whereas symptoms such as daytime sleepiness (physical) may impair mood (emotional), affecting daily functioning (e.g., work or household tasks).

In this work, we build upon an existing systematic review [8] and specifically focus on the subset of disease-specific HRQOL PROMs for OSA. Accordingly, we followed the COSMIN guidelines [11, 26] to enable comparability with formerly published results. Our aim is to identify all HRQOL PROMs for OSA and assess their psychometric properties as well as the feasibility and interpretability of new PROMs that have been developed since then.

## Methods

### Eligibility criteria

This systematic review and this article were conducted in accordance with the PRISMA reporting guidelines. We included all studies that examined adult OSA patients in a PROM development or validation study that aimed to assess the quality of MP in the original language of PROMs. The PROMs needed to focus on HRQOL and had to be fully accessible in English or German. Instruments focused on a single dimension (e.g., only sleepiness scales) were excluded because they do not capture the heterogeneous nature of HRQOL according to our working definition. Further exclusion criteria were PROMs used as a secondary outcome (e.g., studies with HRQOL PROMs as secondary outcomes in pharmaceutical studies that do not assess MP) and those developed for restricted demographics (e.g., elderly or paediatric population, female only, etc.) to be able to cover the broad spectrum of sleep medicine patients. Cross-cultural validation studies were excluded because they focused on original language instruments without potential confounders from translation effects. A comprehensive list of inclusion and exclusion criteria is presented in Table 1.

**Table 1:** Inclusion and exclusion criteria for the systematic search

Inclusion	Exclusion
Development or validation study	Cross-cultural study
PROM measurement properties as outcome	PROM as secondary outcome
HRQOL PROM	PROM with focus on limited domains
Adult OSA patients	Limited demographic sample (e.g. focus on only paediatric patients)
English or German language	
Full text availability	

PROM = patient-reported outcome measures; HRQOL = health-related quality of life.

### Search strategy

Using distinct search strategies for each database (Supplementary Information 1–3), MESH and Emtree terms, if available, and search blocks provided by Terwee et al. [27] for MP, the Medline, Embase and Cochrane databases were systematically searched on June 10, 2024. We did not apply a time or language limit to at least be aware of and report PROMs developed in languages other than English or German. Additionally, we performed a reference search of the included and relevant publications and ensured that our list incorporated all relevant publications listed in the initial review by Abma et al. [8] that fit our inclusion and exclusion criteria. The screening and selection of the studies were performed independently by the authors AW and MB via the web application Rayyan (https://www.rayyan.ai/). For each study, titles and abstracts were screened manually to determine eligibility. If studies were excluded, reasons had to be provided. Comments were left on ambiguous studies that needed discussion. Conflicts were resolved among the reviewers, without the need for an additional mediator.

### COSMIN methodology

Data extraction was performed via templates 2–10 provided in the COSMIN guidelines for systematic reviews [11] by AW and examined by MB. We extracted all the available MPs of all the new studies, which included all the information on *reliability*, *validity*, *responsiveness*, *interpretability* and *feasibility* according to the COSMIN taxonomy [28], where reliability describes the degree to which a measurement is free of measurement errors. It is subdivided into *internal consistency*, *reliability* and *measurement error*, which describe the interrelatedness of items, the degree of true variance of a measurement between patients and the degree of random or systematic error of scores not attributed to true changes in patients in the construct, respectively. Validity comprises content, structural, construct and criterion validity. *Content validity* refers to how adequately the instrument measures the intended construct, which is deemed the most important MP, demanding patient and medical staff involvement. *Structural validity* refers to the PROM score being an adequate reflection of the construct dimensionality. *Construct validity* portrays how adequately instruments are concordant or discordant with similar or different instruments as well as the assessment of relevant group differences. *Criterion validity* most commonly refers to how well instruments reflect a gold standard. In the case of PROMs, this usually refers to the long version of a PROM. *Responsiveness* is an instrument with the ability to detect change over time in a measured construct through the use of change scores. Interpretability and feasibility are not MP; however, they are crucial when searching for the most suitable PROM. *Interpretability* mainly focuses on scores for quantitative interpretation, specifically the minimal important change (MIC) and minimal important difference (MID) and relevant floor and ceiling effects, whereas *feasibility* primarily focuses on the ease of application, time investment and comprehensibility of the instrument. A thorough description of each MP and its most suitable statistical and methodological approaches are described in the initial publication [29] and the manual [11]. Furthermore, we collected data on the PROM items, domain numbers and patient characteristics.

### Risk of bias assessment and quality of measurement properties

We used the COSMIN methodology for the assessment of the risk of bias of selected studies [11]. Initially, we used the old COSMIN manual for systematic reviews [26] and its corresponding checklists for the assessment of the risk of bias of content validity [30] and the remaining MP. Manual 2.0 was updated in August 2024 and introduced some changes, for which we adjusted to ensure that our ratings were up-to-date. The assessment operates according to the “worst score counts” method (“very good”, “adequate”, “doubtful” or “inadequate”). If, e.g., the rating for one item on internal consistency assessment was assigned “doubtful”, even though the majority was “very good”, the overall rating on internal consistency for that PROM would result in “doubtful”. In conclusion, the risk of bias assessment addresses and evaluates the use and of recommended methodological and statistical procedures and the potential bias that may arise if these procedures are not followed or applied accordingly. A comprehensive list with examples can be found in the COSMIN manual on p. 97–216 [11].

After the risk of bias assessment, the quality of the MPs is rated after careful extraction against the COSMIN criteria for good measurement properties, which are found in the manual on pages 55–56 [11]. PROM development and content validity, which both affect MP content validity, are assessed in terms of *comprehensiveness, comprehensibility and relevance,* meaning that the included items fully reflect the intended concept, are suitable for the target population and context, and are clearly understood as intended without missing any key aspects. Structural validity is usually assessed by *confirmatory or exploratory factor analysis or item response theory*. The factor structure is adequate if item loading is sufficient (> 0.30), overlap is minimal, explained variance is ≥ 50% and aligns with the predefined construct structure and model fit indices (e.g., comparative fit index, > 0.95 or root mean square error of approximation < 0.06). Next, internal consistency requires a *Cronbach’s alpha per unidimensional* scale of ≥ 0.70. For reliability, the *intraclass correlation coefficient (ICC)* or *Spearman/Pearson correlation* for stable patients should be ≥ 0.70. For the measurement error rating, the *smallest detectable change (SDC)* and the *limits of agreement need* to be smaller than the MIC. Criterion validity is the *testing of a PROM against an existing gold standard* with a correlation or area under the curve of ≥ 0.70, whereas construct validity comprises *hypothesis testing* by comparing the PROM with similar or dissimilar tools or by conducting group comparisons, where ≥ 0.75% of predefined hypotheses need to hold true. The same applies to responsiveness, where the *confirmed hypotheses* are ≥ 0.75% or the area under the curve is ≥ 0.70%.

### Evidence

Since only single studies per measurement property (MP) per chosen PROM were available, data pooling was not possible. Nevertheless, we applied the *GRADE* approach to summarize and rate the quality of evidence for each PROM as “high”, “moderate”, “low”, or “very low”. Downgrading was based on risk of bias, imprecision (sample sizes < 100 or < 50 if the MP assessment did not already involve sample size), and indirectness (no assessment in the target population). Inconsistency was not considered, as advised in the manual, owing to the lack of study diversity. A comprehensive description of the GRADE approach with examples can be found in the COSMIN manual on p. 57–64 [11].

## Results

**Fig. 1:**
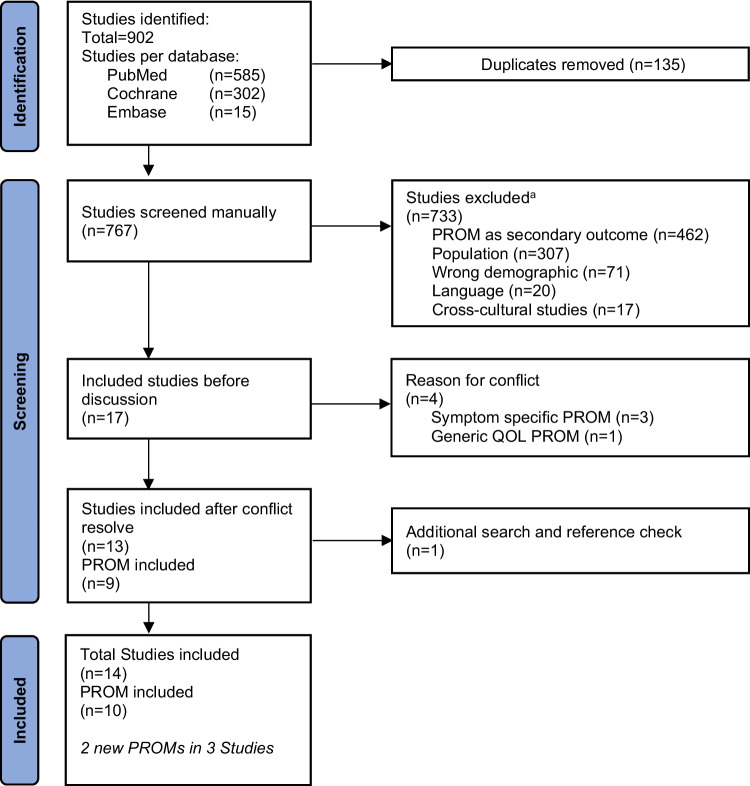
Flow chart of the systematic literature search of Health-Related Quality of Life Patient Reported Outcome Measures (PROM). a = Multiple exclusion reasons were sometimes applied to one study, thus the total number does not add up to 733. Only the 5 most frequent exclusion reasons are displayed. Source: PRISMA 2020 flow diagram — PRISMA statement (prisma-statement.org) under correct use of: Deed—Attribution 4.0 International—Creative Commons, downloaded an modified on 18.09.2024

A total of n = 902 studies were found. After n = 135 duplicates were resolved, the titles and abstracts of 767 studies were screened for eligibility, resulting in n = 13 studies and n = 9 HRQOL PROMs after conflict resolution. The most common exclusion criterion was *PROMs used as a secondary outcome* (n = 462). *Population* (n = 307), describing the focus on a different health disorder within a study, was the second most common reason, followed by exclusion due to *wrong demographic*, e.g., age restrictions (n = 71), non-English studies (n = 20) and cross-cultural studies (n = 17, Supplementary Information 4). The OSAPOSI study was found by reference and added, thus resulting in a total of n = 10 PROMs in 14 studies, including two new PROMs evaluated in three studies since the last review in 2016, where a total of n = 8 PROMs in n = 11 studies were found [8]. The eight PROMs formerly identified are the FOSQ [9], FOSQ-10 [10], which is a short version of the FOSQ; the SAQLI [12, 31, 32]; the QSQ [13]; the MOSAS [14]; the OSAPOSI [15]; the symptom of nocturnal obstruction and related events-25 (SNORE25) [7]; and the visual analogical well-being scale (VAWS) [33]. Figure 1

### New PROMs

We identified two new PROMs since the 2016 review, both of which underwent a full assessment of their psychometric properties. The characteristics of the *P*atient-*R*eported *A*pnea *Q*uestionnaire (PRAQ) [34, 35] and the STAMP instrument, short for its dimensions ‘*S*ymptoms, *T*iredness, *A*lertness, *M*ood and *P*sychosocial’ [36] are displayed in Table 2. The PRAQ was validated in two studies, whereas the STAMP was tested in a single study. The development of the PRAQ was described first [34], followed by nearly complete psychometric validation [35]. This self-report questionnaire takes approximately 15 min to complete and was developed for use in clinical practice. The PROM was originally tested on a Dutch sample, but a translation into the English language exists. It contains ten domains (*symptoms at night, sleepiness, tiredness, daily activities, unsafe situations, memory and concentration, quality of sleep, emotions, social* and *health concerns*) with initially 43 items and 40 items after item reduction and completion. The PRAQ provides a short definition of the terms PROM and HRQOL and utilizes the conceptual model of Wilson and Cleary [19] for the formation of domains and separation of items. The definition aligns with our working definition but remains rather vague in conformity with the status quo. The STAMP was developed and tested on an English-speaking population. It consists of five domains (*symptoms, tiredness, alertness, mood and psychology*) and twelve items. This self-report questionnaire takes approximately three minutes to complete and was developed for use in clinical practice. The STAMP provides a definition of the term HRQOL as well, fitting into our understanding of the term, yet does not mention any kind of HRQOL model as a conceptional basis.

**Table 2:** Characteristics of the PRAQ and STAMP

PROM	Year	Language	Domains (Items)	Administration	Use	Time	Sample Size	Age	Female (%)
*PRAQ*^*1*^	2017	Dutch	10 (43)	self-report	clinical routine	15 min	35 patients 30 medical staff	60–69 50–59	29 53
*PRAQ*	2018	Dutch	10 (40)				180 baseline 105 test–retest 53 follow-up	50.1^a^ 50.4^a^ 55.8^a^	31.7 38.1 25
*STAMP*^*2*^	2019	English	5 (12)	self-report	clinical routine	3 min	10 importance ranking 73 OSA patients 32 healthy subjects	N/A N/A N/A	N/A N/A N/A

1 = Patient-Reported Apnea Questionnaire.

2 = Symptoms, Tiredness, Alertness, Mood and Psychosocial instrument.

a= mean values.

### Risk of bias

The risk of bias ratings for the PRAQ and STAMP are displayed in Table 3. Both PROMs have not been fully validated with respect to the COSMIN standards; however, the PRAQ displays nearly complete validation in two studies, whereas the STAMP shows preliminary results. The most important MP, content validity, was only assessed in the PRAQ development study but lacked a separate confirmatory content validity study. The development and pilot testing of the PRAQ show strong positive evidence for good content validity with mainly “very good” ratings, including input from both patients and medical staff, with two rounds of cognitive validation and an expert survey. Owing to the lack of description of the interview guideline and the transcription process, we gave it an overall “adequate” rating. The PRAQ does not directly involve patients for item generation but uses pooled items of the SAQLI, QSQ and MOSAS, which are created with patient input. Patients and medical staff evaluated the suitability, comprehensiveness, comprehensibility and relevance of these items. The STAMP does not address patients’ comprehensibility or whether recall, response options or items were thoroughly discussed; therefore, it was given a “doubtful” rating. Structural validity was rated “doubtful” for both PROMs. The PRAQ uses a principal component analysis (PCA), where decision criteria and item selection due to formative and reflective models as well as methodological aspects such as rotation and factor loading are sufficiently defined. The domains “Symptoms at night” and “social interaction” were excluded from the PCA due to clinometric formation, meaning they do not necessarily capture the same latent construct, resulting in 33 items included in PCA. However, the PCA is inferior to exploratory and confirmatory factor analysis, leading to downgrading. The STAMP presumably uses confirmatory analysis since a concrete hypothesis on factor loading was tested (“This was completed through a factor analysis, which tested the hypothesis that all variables would load onto one factor” [36]), yet methodological details were not addressed; hence, we downgraded it to “doubtful”. Internal consistency was “very good” for the PRAQ, with Cronbach’s alpha calculated for all unidimensional subscales, while the STAMP displays a one-factor solution; however, five dimensions were formed without reasonable explanation. However, the hypothesized one-factor solution was confirmed, and Cronbach’s alpha was calculated, leading to a “very good” rating. Reliability was rated “doubtful” for the PRAQ and “inadequate” for the STAMP because the former did not provide sufficient proof for stability in patients, whereas the latter did not calculate the ICC but performed a paired t test. The PRAQ performed “adequate” on hypothesis testing regarding convergent and discriminant validity. The downgrading was due to the use of the Patient-Reported Outcomes Measurement Information System (PROMIS) for convergent validity, which was not used and validated on an OSA sample at that time; therefore, it was questionable whether it applied to the target population. The STAMP performed a sensitivity and specificity analysis on OSA patients and healthy controls without a sufficient description of the population’s characteristics. While the PRAQ proved to be quite responsive, it was downgraded to “adequate” owing to the same issue with the PROMIS questionnaire on hypothesis testing. The STAMP did not assess responsiveness. Measurement error was considered “doubtful” for the PRAQ. The PRAQ did not provide sufficient information on the formula used for the calculation of the SEM, and the STAMP did not calculate the required statistics.

**Table 3:** Risk of bias* of measurement properties per included PROM

PROM	Content Validity		Structural Validity		Internal Consist	Reliability	Criterion Validity	Hypothesis testing				Responsiv	Measur Error
	*PROM* *Develop*	*Content Validity Study*						*Converg. Validity*	*Known Group Val*	*Discrim. Validity*			
PRAQ^1^ *Abma *et al*. 2017*	adequate^a^	N/A											
PRAQ *Abma *et al*. 2018*			doubtful^l^		very good	doubtful^c^	N/A	adequate^d^	N/A	adequate^d^		adequate^e^	doubtful^f^
STAMP^2^ *Mehta *et al*. 2019*	doubtful^g^	N/A	doubtful^h^		very good	inadequate^i^	N/A^j^	N/A	doubtful^k^	N/A		N/A	N/A

* = The COSMIN Risk of bias assessments operates according to the “worst score counts” principle. More than one downgrade might apply, yet we decided to depict just one

1 = Patient-Reported Apnea Questionnaire.

2 = Symptoms, Tiredness, Alertness, Mood and Psychosocial instrument.

a=Assumable that interview guideline exist and interviews were recorded and transcribed

b=Principal component analysis was used instead of confirmatory or exploratory factor analysis

c=No evidence that no systematic change has occurred (e.g. mean change between t0 and t1 or Bland–Altman-Plot) was presented

d=Patient Reported Outcomes Measurement Information System (PROMIS) was not yet validated in an OSA sample, yet shows relevant domain overlap to PRAQ

e=See d.

f=See c.

g=No clear if patients were asked about comprehensibility of items, response options and recall period.

h=No explanation of methodology (e.g. rotation method), presumably confirmatory factor analysis since hypothesis was tested that all items load on one factor

i=Paired t-test was calculated instead of intraclass correlation coefficient (ICC) or correlation

j=Correlation with Apnea–Hypopnea-Index or Flow Limitation Index calculated, but is not an adequate reflection of Quality of Life but rather a diagnostic standard for severity of obstructive sleep apnea.

k=No description of characteristics of subgroup

### Quality of measurement properties

The qualities of the measurement properties are displayed in Table 4, which presents ratings based on the COSMIN criteria for good measurement properties [11]. The PRAQ met the quality criteria in almost every category. Only one positive to indeterminate rating for structural validity was assigned since multiple factor loadings were observed for more than ten percent of all the items, yet the loadings were all less than 0.3, which is considered sufficiently small. For measurement error, only the SEM was calculated, and other important statistics for the assessment of existing measurement error were missing. Overall, the STAMP does not provide the needed requirements set by the COSMIN standard or calculate different statistics in most cases, e.g., reliability was displayed via a t-test and not the ICC. It appears that criterion validity was assessed for the STAMP since the AHI and Flow Limitation Index were correlated with the STAMP; however, both are not valid reflections of HRQOL but rather important physiological metrics; therefore, they cannot be considered valid gold standards.

**Table 4:** Quality of measurement properties per included PROM

PROM	Content Validity		Structural Validity	Internal Consist	Reliability	Criterion Validity	Hypothesis testing			Responsiv		Measur. Error
	*PROM* *Develop*	*Content Validity Study*					*Converg. Validity*	*Known Group Val*	*Discrim. Validity*			
PRAQ^1^ *Abma *et al*. 2017*	+	N/A										
PRAQ *Abma *et al*. 2018*			+?^a^	+	+	N/A	+	+	+	+		?^b^
STAMP^2^ *Mehta *et al*. 2019*	?^c^	N/A	?^d^	+	?	N/A	N/A	-^e^	N/A	N/A		N/A

1 = Patient-Reported Apnea Questionnaire.

2 = Symptoms, Tiredness, Alertness, Mood and Psychosocial instrument.

a = Three items displayed in PCA load on more than 1 factor (ca. 15%) yet below 0.3 loading

b = Limits of agreement, minimal important change and smallest detectable change not calculated

c = No assessment of comprehensibility, recall period, response options etc.

d = No comparative fit index or Tucker-Lewis index calculated.

e = No hypothesis formed for subgroups (t-test and sensitivity and specificity were tested)

Furthermore, we evaluated the PRAQ and STAMP for feasibility and interpretability (see Appendix Table 5 and 6) on the basis of characteristics reported in the literature. Compared with previously recommended PROMs with good evidence for content validity, the PRAQ (10 domains, 40 items) requires approximately 15 min to complete. The STAMP (5 domains, 12 items) demonstrated superior feasibility, requiring only 3 min to complete. In terms of interpretability, the PRAQ exhibited a floor effect on the sleepiness item. In contrast, the STAMP initially showed multiple floor and ceiling effects, leading to the exclusion of affected items from the final version. Neither PROM provides cut-off scores nor establishes minimal important change (MIC) or minimal important difference (MID) values. However, the PRAQ introduces a patient-friendly, smiley-based reporting system that visualizes individual item and domain scores to facilitate clinical interpretation. The PRAQ uses a 7-point Likert scale per item, with response options varying by domain (e.g., problem intensity, effort, or prevalence), where higher scores indicate greater severity or worse HRQOL. The maximum total score is 280 points, reflecting the worst possible outcome. The STAMP uses a 5-point scale (ranging from “never” to “always”), with higher scores indicating worse HRQOL and a maximum total score of 60 points.

*Evidence*

Due to the low number of studies, statistical pooling was not possible, but we assessed the overall rating via the COSMIN GRADE [11] approach (Supplementary Information 5–6). When applying the criteria, we found the PRAQ to be of overall moderate quality. This is to be expected owing to the availability of a single study per MP, causing at least one serious downgrade if the risk of bias was at least adequate. Responsiveness, even though rated as adequate in the risk of bias assessment, was downgraded for imprecision, since the sample size was less than n = 100. The STAMP displays overall low to very low evidence, mainly because of very serious downgrading due to risk of bias. Some MPs were not assessed and therefore not graded.

## Discussion

In this updated review, we aimed to identify HRQOL PROMs for OSA and conducted a psychometric assessment of those developed since the 2016 systematic review [8]. We found two such PROMs: the PRAQ, which was developed in two studies [34, 35], is a thoroughly validated PROM of moderate evidence, showing some limitations with respect to structural validity, reliability and measurement error. The PRAQ demonstrates strong PROM development, comparable to the PROMs recommended in the 2026 review—namely, SAQLI, QSQ, OSAPOSI, and MOSAS—and actively incorporates items from three of those PROMs (SAQLI, MOSAS, and QSQ—the OSAPOSI is not available, which likely cause the exclusion). The process of item selection and domain creation followed well-established standards, including patients’ and medical staff’s perspectives. For the STAMP, which was developed in one study [36], only preliminary results exist, and those are predominantly of lower evidence with doubtful or inadequate ratings of the risk of bias assessment. Important aspects of the validation phase were either not addressed or different methods were used for the assessment of MP, deviating from the COSMIN recommendations. Compared with the PROMs evaluated in the previous review, the PRAQ can be considered among the recommended instruments, demonstrating good content validity, an acceptable risk of bias, and strong measurement property quality. In contrast, the lower level of evidence for the STAMP aligns with that of most PROMs developed for OSA in the previous review. Both PROMs show decent feasibility, with the STAMP taking less time to complete, thus decreasing patient burden when administered. The PRAQ, with a completion time of approximately 15 min, takes slightly more time than the MOSAS (6 domains, 23 items) and QSQ (5 domains, 32 items) but considerably less than the SAQLI (5 domains, 84 items). Neither cut-off scores nor MICs or MDIs are available for both PROMs, which reduces the overall interpretability; however, the PRAQ offers a visual tool that displays individual scores and domain scores for a convenient overview. Notably, the PRAQ had little effect on patient-centeredness and was less useful to clinical staff but showed overall good acceptance by patients [37].

When administering a PROM, it is important to consider its potential and limitations. Thus, it is not always a global score but rather a single item that can provide information about a patient's most serious problems. Similarly, not every patient necessarily needs a battery of PROMs that are not adequately discussed during a doctor’s visit and fail to be readministered to detect changes after therapy implementation [38]. Both PROMs give a short definition of HRQOL, but the PRAQ also specifies the term PROM and accounts for conceptional frameworks of HRQOL in their development process. We placed particular emphasis on this aspect because, as discussed above, the field remains characterized by a multitude of definitions. A clear specification of terms is therefore helpful for understanding the underlying epistemological interest of a PROM’s developer and might ease contextualizing the construct being measured. This allows for identifying whether one’s own and the presented definition are congruent or aligned with the broader scientific narrative [21], which is the reason why we establish a working definition within this review.

Disease-specific PROMs can help uncover key patient concerns—often including sensitive areas such as sexual health or emotional and social trouble— and can further support the targeted use of symptom- or function-specific instruments or help initiate better patient‒physician communication. While generic tools allow for cross-condition comparability, disease-specific instruments are often more appropriate in the context of patient-centric care. It has been suggested that disease-specific HRQOL PROMs are particularly valuable in clinical practice because of their greater responsiveness, face validity, and ability to capture meaningful changes in individual patients, whereas generic PROMs, although less sensitive to specific conditions, may be useful for comparisons across different patient populations and for informing organizational- or system-level assessments [39]. Evidence indicates that disease-specific PROMs are more effective in improving outcomes on the micro level (patient-clinician encounter) with 71% of studies reporting benefit, compared to 38% for generic or combined PROMs. This highlights the key role for outcomes such as symptom burden, HRQOL, physical and mental functioning etc. [40]. Moreover, the implementation of HRQOL instruments may enhance awareness among physicians and clinical staff for HRQOL of patients and might foster more comprehensive diagnostic assessment. This, in turn, can contribute to more detailed and higher-quality medical documentation, enabling a more holistic characterization of the patient’s condition [41]. Even widely used tools such as the PROMIS show limited sensitivity in capturing treatment effects in patients with OSA [42], stressing the importance of disease-specific tools.

### Limitations

The first major limitation regarding the evidence is the small number and size of included studies, limiting precision and making statistical pooling impossible, since only two studies for the PRAQ and one for the STAMP are available. Second, in 2024, the COSMIN group released an updated manual for the assessment of measurement properties [11], changing some criteria for the risk of bias assessment and the quality assessment for MP. When applying the old standard, the rating of the STAMP did not change between both manuals, yet the PRAQ, e.g., would have had a structural validity and measurement error rating of “adequate”, since it was less strict on the method, whereas the new standard recommended a rating of “doubtful” if a PCA was performed. Similarly, reliability and measurement error was downgraded from “very good” to “adequate”, since the new manual explicitly requires a description of the specific statistics, e.g. whether ICC_agreement_ (very good) or the ICC_consistency_ (adequate to doubtful) was used [11, 26]_._ This aggravates a comparison between the old and current review, placing the PRAQ lower than the rating system of the former review. A full re-rating of previously recommended PROMs under COSMIN 2.0 would have strengthened direct comparability, but this was beyond the predefined scope of the present update review, which specifically focused on PROMs emerging after 2016. Similarly, the STAMP references Abma et al. but was not fully assessed according to COSMIN, limiting comparability, which might influence the evidence rating. Third, interpretability is limited because of missing cut-off, MIC, and MID values in both PROMs, although the visual tool of the PRAQ appears suitable for clinical use. In practice, HRQOL instruments should primarily support identifying affected areas and facilitating communication, not necessarily defining strict thresholds, which are essential in clinical trials. Finally, COSMIN’s rigorous criteria may underrate PROMs with otherwise acceptable evidence.

A key limitation of the review process is the narrow definition of the inclusion and exclusion criteria, reflecting our specific research focus. We specifically looked at development studies and did not consider cross-cultural validation studies, which might provide important insight into the validity and psychometric properties of some PROMs. In any case, those did not exist for the two new PROMs. We refrained from rerating previously rated PROMs, which may limit comparability due to updates in the COSMIN manual and interrater differences. Finally, subjective judgment in the assessment process and language limitations (English and German only) may have led to the exclusion of relevant instruments.

## Conclusion

Our results indicate that the PRAQ provides strong evidence among disease-specific HRQOL PROMs for patients with OSA and holds a similar level of recommendation as the SAQLI, QSQ, MOSAS, and OSAPOSI. It occupies a distinct position and due to its explicit integration of items from three of these instruments (SAQLI, QSQ, and MOSAS), effectively functioning as a composite PROM. In contrast, the STAMP instrument demonstrates overall low-quality evidence according to COSMIN standards and would benefit from further validation. The selection of a PROM remains highly context dependent, influenced by factors such as available resources, clinical priorities, and intended application (clinical practice vs. research; diagnosis vs. monitoring). With the increasing emphasis on patient-centred care in current regulatory guidelines, the routine assessment of HRQOL is likely to become standard practice. For future research, it is recommended to prioritize the clinical utility of high-quality PROMs, systematically evaluating patient benefit as well as implementation feasibility and staff burden, while leveraging PROMs to identify often-overlooked concerns—such as emotional, functional, or sensitive topics—and incorporate these insights into the clinician–patient interaction.

## Electronic supplementary material

Below is the link to the electronic supplementary material.

Supplementary file1 (PDF 359 KB)

## Data Availability

All additional data are available upon reasonable request from the first author.

## Appendix

Table 5

**Table 5:** Feasibility of the PRAQ and STAMP

Feasibility	PRAQ^1^	STAMP^2^
Type of administration	Self-report	Self-report
Use	Clinical routine	Clinical routine
Completion time	15 min	3 min
Languages	Dutch (English translation available)	English
Availability	Available on request	Available in publication
Number of domains and items	10,43	5,12
Domains	1 = Symptom at night; 2 = Sleepiness, 3 = Tiredness; 4 = Daily activities; 5 = Unsafe situations; 6 = Memory and concentration; 7 = Quality of sleep; 8 = Emotions; 9 = Social; 10 = Health concerns	1 = Symptoms, 2 = Tiredness 3 = Alertness, 4 = Mood 5 = Psychological
Scale	7-point-likert (problem intensity, effort, or prevalence; higher scores = worse HRQOL)	6-point-scale (0 = never; 5 = always; higher scores = worse HRQOL)

1= Patient-Reported Apnea Questionnaire

2= Symptoms, Tiredness, Alertness, Mood and Psychosocial instrument

Table 6

**Table 6:** Interpretability of the PRAQ and STAMP

Interpretability	PRAQ^1^	STAMP^2^
Floor Effects	on "sleepiness" item (20% lowest category 1–1.5)	1 item, but removed from PROM
Ceiling Effects	N/A	5 items, but removed from PROM
Items with missing values	no missings allowed in computer version, but 1 missing item on paper version	N/A
Cut-off score	N/A, visual smiley-based tool	N/A
MIC/MID	N/A	N/A

1= Patient-Reported Apnea Questionnaire

2= Symptoms, Tiredness, Alertness, Mood and Psychosocial instrument

## Abbreviation

AHI: Apnea-hypopnea index
COSMIN: COnsensus based Standards for the selection of health Measurement
FOSQ: Functional Outcome of Sleep Questionnaire
HRQOL: Health-related quality of life
ICC: Intraclass correlation coefficient
MIC: Minimal important change
MID: Minimal important difference
MOSAS: Maugeri Obstructive Sleep Apnea
MP: Measurement properties
OSA: Obstructive sleep apnea
OSAPOSI: Obstructive Sleep Apnea Patient-Oriented Severity Index
PCA: Principal component analysis
PRAQ: Patient-Reported Apnea Questionnaire
PROM: Patient-reported outcome measures
PROMIS: Patient-Reported Outcomes Measurement Information System
PSG: Polysomnography
QOL: Quality of life
QSQ: Quebec Sleep Questionnaire
SAQLI: Calgary Apnea Quality of Life Index
SDC: Smallest detectable change
SEM: Standard error of measurement
STAMP: Symptoms, Tiredness, Alertness, Mood and Psychosocial instrument

## Declarations

### Registration and review protocol

This systematic review was registered in the international prospective register of systematic reviews (PROSPERO) under the registration number: CRD42024602020.
