# Supplementary material for: Health-related quality of life in patients with obstructive sleep apnea – a systematic review update assessing the quality of measurement properties of patient-reported outcome measures
Source: Sleep Breath. 2026 Apr 21;30(2):132. doi: 10.1007/s11325-026-03676-1 (PMC13099707; doi:10.1007/s11325-026-03676-1)
Supplement: Supplementary file 1 — Supplementary file1 (PDF 359 KB) [file 11325_2026_3676_MOESM1_ESM.pdf]

Supplementary Information

Title: Health Related Quality of Life in patients with Obstructive Sleep Apnea – A Systematic Review update assessing the Quality of Measurement Properties of Patient-Reported Outcome Measures

Journal: Sleep and Breathing

Authors

M.A. Alina Wildenauer[1], alina.wildenauer@rlk.uk-essen.de

Prof. Dr. med. Christoph Schöbel[1], christoph.schoebel@rlk.uk-essen.de

Dr. rer. nat. Torsten Eggert[1], torsten.eggert@rlk.uk-essen.de

Dr. rer. medic. Marcel Braun[1], marcelbraun@mac.com

Affiliation1: University Medicine Essen, Ruhrlandclinic – West German Lung Centre, Centre for Sleep and Telemedicine, Essen, Germany

Supplementary Information 1: PubMed Search Strategy with 585 hits on June 10, 2024

Category	Search terms
Concept	Quality of life[tiab] OR QoL[tiab] OR QOL[tiab] OR HRQoL[tiab] OR HRQL[tiab] OR HRQOL[tiab] OR Health-Related Quality of Life[tiab] OR Health Related Quality of Life[tiab] OR Quality of life[MeSH]
Population	Sleep apnea[tiab] OR sleep apnoea[tiab] OR Obstructive sleep disorder*[tiab] OR OSA[tiab] OR OSAS[tiab] OR Obstructive sleep apnea [tiab] OR "Sleep Apnea Syndromes"[MeSH] OR "Sleep Apnea, Obstructive"[Mesh]
Type of Instrument	patient-reported outcome measure*[tiab] OR Patient Reported Outcome Measure*[tiab] OR PROM[tiab] OR PROMs[tiab] OR questionnaire*[tiab] OR survey[tiab] OR surveys[tiab] OR instrument*[tiab] OR scale[tiab] OR scales[tiab] OR checklist*[tiab] OR assessment*[tiab] OR computer adaptive test[tiab] OR computer adaptive tests[tiab] OR self-report*[tiab] OR diary[tiab] OR diaries[tiab] OR log [tiab] OR logs OR interview*[tiab] OR "Patient Reported Outcome Measures"[Mesh] questionnaires[MeSH] OR self-report [MeSH] OR interviews as topic [MeSH]
Measurement Propertie[a]	“Validation Study”[pt] OR “Comparative Study”[pt] OR “psychometrics”[MeSH] OR psychometr*[tiab] OR clinimetr*[tw] OR clinometr*[tw] OR “ patient outcome assessment”[MeSH] OR “outcome assessment”[tiab] OR “outcome measure*”[tw] OR “observer variation”[MeSH] OR “observer variation”[tiab] OR “Health Status Indicators”[MeSH] OR “reproducibility of results”[MeSH] OR reproducib*[tiab] OR “discriminant analysis”[MeSH] OR reliab*[tiab] OR unreliab*[tiab] OR valid*[tiab] OR “coefficient of variation”[tiab] OR coefficient[tiab] OR homogeneity[tiab] OR homogeneous[tiab] OR “internal consistency”[tiab] OR cronbach*[tiab] AND alpha[tiab] OR alphas[tiab] OR item[tiab] AND correlation*[tiab] OR selection*[tiab] OR reduction*[tiab] OR agreement[tw] OR precision[tw] OR imprecision[tw] OR “precise values”[tw] OR test-retest[tiab] OR test[tiab] AND retest[tiab] OR reliab*[tiab] AND test[tiab] OR retest[tiab] OR stability[tiab] OR interrater[tiab] OR inter-rater[tiab] OR intrarater[tiab] OR intra-rater[tiab] OR

	intertester[tiab] OR inter-tester[tiab] OR intratester[tiab] OR intra-tester[tiab] OR interobserver[tiab] OR inter-observer[tiab] OR intraobserver[tiab] OR intra-observer[tiab] OR intertechnician[tiab] OR inter-technician[tiab] OR intratechnician[tiab] OR intra-technician[tiab] OR interexaminer[tiab] OR inter-examiner[tiab] OR intraexaminer[tiab] OR intra-examiner[tiab] OR interassay[tiab] OR inter-assay[tiab] OR intraassay[tiab] OR intra-assay[tiab] OR interindividual[tiab] OR inter-individual[tiab] OR intraindividual[tiab] OR intra-individual[tiab] OR interparticipant[tiab] OR inter-participant[tiab] OR intraparticipant[tiab] OR intra-participant[tiab] OR kappa[tiab] OR kappa's[tiab] OR kappas[tiab] OR repeatab*[tw] OR replicab*[tw] OR repeated[tw] AND measure[tw] OR measures[tw] OR findings[tw] OR result[tw] OR results[tw] OR test[tw] OR tests[tw] OR generaliza*[tiab] OR generalisa*[tiab] OR concordance[tiab] OR intraclass[tiab] AND correlation*[tiab] OR discriminative[tiab] OR "known group"[tiab] OR "factor analysis"[tiab] OR "factor analyses"[tiab] OR "factor structure"[tiab] OR "factor structures"[tiab] OR dimension*[tiab] OR subscale*[tiab] OR multitrait[tiab] AND scaling[tiab] AND analysis[tiab] OR analyses[tiab] OR "item discriminant"[tiab] OR "interscale correlation*"[tiab] OR error[tiab] OR errors[tiab] OR "individual variability"[tiab] OR "interval variability"[tiab] OR "rate variability"[tiab] OR variability[tiab] AND analysis[tiab] OR values[tiab] OR uncertainty[tiab] AND measurement[tiab] OR measuring[tiab] OR "standard error of measurement"[tiab] OR sensitiv*[tiab] OR responsive*[tiab] OR limit[tiab] AND detection[tiab] OR "minimal detectable concentration"[tiab] OR interpretab*[tiab] OR minimal[tiab] OR minimally[tiab] OR clinical[tiab] OR clinically[tiab] AND important[tiab] OR significant[tiab] OR detectable[tiab] AND change[tiab] OR difference[tiab] OR small*[tiab] AND real[tiab] OR detectable[tiab] AND change[tiab] OR difference[tiab] OR "meaningful change"[tiab] OR "ceiling effect"[tiab] OR "floor effect"[tiab] OR "Item response model"[tiab] OR IRT[tiab] OR Rasch[tiab] OR "Differential item functioning"[tiab] OR DIF[tiab] OR "computer adaptive testing"[tiab] NOT "address"[Publication Type] OR "biography"[Publication Type] OR "case reports"[Publication Type] OR "comment"[Publication Type] OR "directory"[Publication Type] OR "editorial"[Publication Type] OR "festschrift"[Publication Type] OR "interview"[Publication Type] OR "lecture"[Publication Type] OR "legal case"[Publication Type] OR "legislation"[Publication Type] OR "letter"[Publication Type] OR "news"[Publication Type] OR "newspaper article"[Publication Type] OR "patient education handout"[Publication Type] OR "popular work"[Publication Type] OR "congress"[Publication Type] OR "consensus development conference"[Publication Type] OR "consensus development conference, nih"[Publication Type] OR "practice guideline"[Publication Type] NOT "animals"[MeSH Terms] NOT "humans"[MeSH Terms]

a=Modified Search Block by Terwee et al. (2009)
All categories were combined by "AND" command

Supplementary Information 2: Cochrane Search Strategy with 302 hits on June 10, 2024

Category	Search terms
Concept	Quality of life OR QoL OR QOL OR HRQoL OR HRQL OR HRQOL OR Health-Related Quality of Life OR Health Related Quality of Life OR Quality of life<br>Quality of Life[mh]
Population	Sleep apnea OR sleep apnoea OR Obstructive sleep disorder* OR OSA OR OSAS OR Obstructive sleep apnea OR "Sleep Apnea Syndromes" OR "Sleep Apnea, Obstructive"<br>Sleep Apnea Syndromes[mh]
Type of Instrument	patient-reported outcome measure* OR Patient Reported Outcome Measure* OR PROM OR PROMs OR questionnaire* OR survey* OR instrument* OR scale* OR checklist* OR assessment* OR computer adaptive test OR computer adaptive tests OR self-report* OR diary* OR log OR logs OR interview* OR "Patient Reported Outcome Measures" OR questionnaires OR self report OR interviews as topic<br>Patient Reported Outcome Measures [mh]<br>Surveys and Questionnaires[mh]<br>Interviews as Topic[mh]
Measurement Properties[a]	instrumentation OR methods OR “Comparative Study” OR psychometr* OR clinimetr* OR clinometr* OR “patient outcome assessment” OR “outcome assessment” OR “outcome measure*” OR “observer variation” OR “observer variation” OR “Health Status Indicators” OR “reproducibility of results” OR reproducib* OR “discriminant analysis” OR reliab* OR unreliab* OR valid* OR “coefficient of variation” OR coefficient OR homogeneity OR homogeneous OR “internal consistency” OR cronbach* NEXT alpha OR correlation OR item OR selection OR agreement OR precision OR imprecision OR “precise values” OR test-retest OR test NEXT retest OR reliab* OR stability OR interrater OR inter-rater OR intrarater OR intra-rater OR intertester OR inter-tester OR intratester OR intra-tester OR interobserver OR inter-observer OR intraobserver OR intra-observer OR intertechnician OR inter-technician OR intratechnician OR intra-technician OR interexaminer OR inter-examiner OR intraexaminer OR intra-examiner OR interassay OR inter-assay OR intraassay OR intra-assay OR interindividual OR inter-individual OR intraindividual OR intra-individual OR interparticipant OR inter-participant OR intraparticipant OR intra-participant OR kappa OR kappas OR kappas OR repeatab* OR replicab* OR repeated AND measure OR measures OR findings OR result OR results OR test OR tests OR generaliza* OR generalisa* OR concordance OR intraclass AND correlation* OR discriminative OR “known group” OR “factor analysis” OR “factor analyses” OR “factor structure” OR “factor structures” OR dimension* OR subscale* OR multitrait AND scaling AND analysis OR analyses OR “item discriminant” OR “interscale correlation*” OR error OR errors OR “individual variability” OR “interval variability” OR “rate variability” OR variability AND analysis OR values OR uncertainty AND measurement OR measuring OR “standard error of measurement” OR sensitiv* OR responsive* OR limit AND detection OR “minimal detectable concentration” OR interpretab* OR minimal OR minimally OR clinical OR clinically AND important OR significant OR detectable AND change OR difference OR small* AND real OR detectable AND change OR difference OR “meaningful change” OR “ceiling effect” OR “floor effect” OR “Item response model” OR IRT OR Rasch OR “Differential item functioning” OR DIF OR “computer adaptive testing”<br>Validation Study[mh]

	Psychometrics[mh]<br>Outcome Assessment[mh]<br>Health Status Indicators[mh]<br>Reproducibility of Results[mh]<br>Discriminant Analysis[mh]<br>validation stud*:pt<br>comparative stud*:pt

a=Modified Search Block by Terwee et al.(2009)
All categories were combined by "AND" command

Supplementary Information 3: Embase Search Strategy with 15 hits on June 10, 2024

Category	Search terms
Concept	'quality of life':ti,ab,kw OR 'health related OR quality of life questionnaire':ti,ab,kw
Population	'sleep apnea syndromes':ti,ab,kw OR 'obstructive OR sleep apnea':ti,ab,kw
Type of Instrument	'patient-reported outcome':ti,ab,kw OR questionnaire:ti,ab,kw OR survey:ti,ab,kw OR scale:ti,ab,kw OR assessment:ti,ab,kw OR 'computer adaptive test':ti,ab,kw OR 'self report':ti,ab,kw OR diary:ti,ab,kw OR interview:ti,ab,kw
Measurement Properties	'validation study'/exp OR 'validation study'

All categories were combined by "AND" command

Supplementary Information 4: Translation and Cross-cultural validation studies of selected PROMs

PROM	Article	Methodology	Language
SAQLI[a]	Balsevicius et al. 2008	Translation and cross-cultural validation	Lithuanian
	Hu et al .2019	Translation and cross-cultural validation	Chinese
	Kasibowska-Kuźniar et al. 2004	Translation and cross-cultural validation	Polish
	Kobayashi et al. 2013	Translation and cross-cultural validation	Japanese
	Mok et al. 2004	Translation and cross-cultural validation	Chinese, Cantonese
	Perrone et al. 2021	Translation, no cross-cultural validation	Portuguese
	Saddki et al. 2013	Translation and cross-cultural validation	Malay
	Rahavi-Ezabadi et al. 2016	Translation and cross-cultural validation	Persian
	Sampaio et al. 2012	Translation and cross-cultural validation,	Portuguese
	Catalán et al. 2012	Translation and cross-cultural validation	Spanish
	Dutt et al. 2013	Translation, no cross-cultural validation	Indian? "vernacular language of patients"
	Jang et al. 2014	Translation and cross-cultural validation	Korean
FOSQ[b]	Banhiran et al. 2012	Translation and cross-cultural validation	Thai
	Büttner et al. 2007	Cross-cultural validation	German
	Kasibowska-Kuźniar et al. 2004	Translation and cross-cultural validation	Polish
	Korpe et al. 2013	Translation and cross-cultural validation	Swedish
	Stavem et al. 2004	Translation and cross-cultural validation	Norwegian
	Ferreret al. 1999	Translation and cross-cultural validation	Spanish
	Izci et.al. 2004	Translation and cross-cultural validation	Turkish
FOSQ-10	Rahavi-Ezabadi et al. 2016	Translation and cross-cultural validation	Iranian/Farsi
	Rey de Castro et al. 2018	Translation and	Spanish

		cross-cultural validation	
QSQ[c]	Catalán et al. 2012	Cross-cultural validation	Spanish
	Huo et al. 2011	Translation and cross-cultural validation	Chinese
	Tavares de Melo Júnior et al. 2017	Translation and cross-cultural validation	Portuguese

SAQLI=Calgary Apnea Quality of Life Index
FOSQ= Functional Outcome of Sleep Questionnaire
QSQ=Quebec Sleep Questionnaire

Supplementary Information 5: GRADE rating of measurement properties of PRAQ[1]

GRADE*	Content Validity		Structural Validity	Internal consistency	Reliability	Criterion Validity	Hypothesis testing			Responsiveness	Measurement Error
	PROM Development	*Content Validity Study*					*Convergent Validity*	*Known Group Validity*	*Discriminant Validity*		
High[a]											
Moderate[b]		N/A				N/A		N/A			
Low[c]											
Very low[d]											

*= GRADE approach by Mokkink et al. 2024 as presented in the Manual for COSMIN guideline for systematic reviews of patient-reported outcome measures version 2.0. [11, p.62] with the ratings high, moderate, low and very low with levels expressing the level of certainty in the evidence (a-d)
1= Patient-Reported Apnea Questionnaire
a= We are very confident that the true measurement property lies close to that of the estimate of the measurement property
b= We are moderately confident in the measurement property estimate: the true measurement property is likely to be close to the estimate of the measurement property, but there is a possibility that it is substantially different
c= Our confidence in the measurement property estimate is limited: the true measurement property may be substantially different from the estimate of the measurement property
d= We have very little confidence in the measurement property estimate: the true measurement property is likely to be substantially different from the estimate of the measurement property

Supplementary Information 6: GRADE* rating of measurement properties of STAMP1

GRADE*	Content Validity		Structural Validity	Internal consistency	Reliability	Criterion Validity	Hypothesis testing			Responsiveness	Measurement Error
	PROM Development	*Content Validity Study*					*Convergent Validity*	*Known Group Validity*	*Discriminant Validity*		
High[a]											
Moderate[b]		N/A				N/A	N/A		N/A	N/A	N/A
Low[c]											
Very low[d]											

*= GRADE approach by Mokkink et al. 2024 as presented in the Manual for COSMIN guideline for systematic reviews of patient-reported outcome measures version 2.0. [11, p.62] with the ratings high, moderate, low and very low with levels expressing the level of certainty in the evidence (a-d)
2= Symptoms, Tiredness, Alertness, Mood and Psychosocial instrument
a= We are very confident that the true measurement property lies close to that of the estimate of the measurement property
b= We are moderately confident in the measurement property estimate: the true measurement property is likely to be close to the estimate of the measurement property, but there is a possibility that it is substantially different
c= Our confidence in the measurement property estimate is limited: the true measurement property may be substantially different from the estimate of the measurement property
d= We have very little confidence in the measurement property estimate: the true measurement property is likely to be substantially different from the estimate of the measurement property
